# MPT64 Protein from *Mycobacterium tuberculosis* Inhibits Apoptosis of Macrophages through NF-kB-miRNA21-Bcl-2 Pathway

**DOI:** 10.1371/journal.pone.0100949

**Published:** 2014-07-07

**Authors:** Qingmin Wang, Shupeng Liu, Ying Tang, Qiuhong Liu, Yongjie Yao

**Affiliations:** 1 Division of Aviation Medicine, Naval medical Research Institute, Shanghai, China; 2 Changhai Hospital, the Second Military medical University, Shanghai, China; University of Padova, Medical School, Italy

## Abstract

MPT64 is one of the secreted proteins from *Mycobacterium tuberculosis*. Little is known about its role in infection by *Mycobacterium tuberculosis*. In this study, we demonstrated that MPT64 could dose-dependently inhibit the apoptosis of RAW264.7 macrophages induced by PPD-BCG. Quantitative real-time PCR results showed that the expression of bcl-2 increased in macrophages treated with MPT64 compared with PPD-treated cells. Furthermore, the results provided strong evidence that bcl-2 up-regulation was positively controlled by miRNA-21. Finally, NF-κB was identified as the transcription factor for miRNA-21 using a ChIP assay. It can be concluded from our study that MPT64 could inhibit the apoptosis of RAW264.7 macrophages through the NF-κB-miRNA21-Bcl-2 pathway.

## Introduction

Infection with *Mycobacterium tuberculosis* (*M.TB*) remains a major cause of morbidity and mortality throughout the world, resulting in 3 million deaths and over 9 million new cases of tuberculosis each year [1]. The increased emergence of multidrug-resistant (MDR) strains of *M.TB* and co-infection with HIV have complicated the threat [2]. Hunting for new strategies to control *TB* requires a better understanding of the complex interactions between the host macrophages and *Mycobacterium tuberculosis*. To escape the host's immune response, *M. tuberculosis* utilizes many strategies to manipulate infected host macrophages. After phagocytosis by macrophages, *Mycobacterium tuberculosis* tries to survive and replicate in macrophages using different strategies that can resist fusion of the phagosome with the lysosome to avoid killing [3]. Recent research has focused on the interactions between host macrophages and *Mycobacterium tuberculosis*.

On one hand, macrophages may undergo apoptosis after the phagocytosis of *Mycobacterium tuberculosis,* which will result in the death of bacilli. Some reports demonstrate that apoptosis is an important way to kill *Mycobacterium tuberculosis* [4]–[6]. Apoptotic macrophages are abundant in human granulomas, and virulent strains of mycobacteria are less apoptogenic than attenuated strains are [7]–[8]. On the other hand, some components of *Mycobacterium tuberculosis* can inhibit the apoptosis of macrophages, which is helpful for the persistence of bacilli. Hence, apoptosis inhibition seems to be a virulence factor that is associated with the up-regulation of anti-apoptotic molecules and down-regulation of pro-apoptotic molecules.

In mycobacteria, several anti-apoptotic components have been identified, including SecA2, Rv3654v, Rv3655c and protein kinaseE (PknE) [9]–[11]. MPT64 is one of the proteins secreted from *Mycobacterium tuberculosis*, but little is known about its role during *Mycobacterium tuberculosis* infection. Here, we report that MPT64 protein from *Mycobacterium tuberculosis* can inhibit the apoptosis of RAW264.7 macrophages in vitro. The mechanism includes the up-regulation of bcl-2, the involvement of increased miRNA21 and the control of the transcription factor NF-κB.

## Materials and Methods

### Culture of RAW264.7 cell lines

RAW264.7 cells were cultured at 37°C and 5% CO_2_ in RPMI1640 supplemented with 10% FBS, streptomycin and penicillin. RAW264.7 cells were induced with phorbol myristate acetate (PMA) (Sigma) at 10 ng/ml final concentration for approximately 24 hours.

### Purification and identification of recombinant GST-tagged MPT64

The mature coding region of mpt64 from *M. tuberculosis* was cloned into the *BamH1* and *Xhol1* sites of plasmid vector pGEX5T. MPT64 protein was produced as a GST-tagged recombinant protein. The recombinant protein was purified from *E. coli k802* by affinity chromatography with Glutathione Sephrose-4B.

To remove the nonspecific effect of LPS, the purified MPT64 protein was treated by polymyxin B (Sigma) according to the manufacturer's instruction. Then, LPS was tested using a Limulus Assay Kit [GenScript (Nanjing) Co].

Purification of MPT64 was confirmed by Coomassie Brilliant Blue R-250 and by immunoblotting using a mAb specific for MPT64 (MyBiosource, San Diego, CA). The recombinant MPT64 protein was transferred onto a nitrocellulose membrane, and the membrane was probed with a 1∶1000 dilution of MPT64-specific antibody and with horseradish peroxide-conjugated secondary antibodies at a dilution of 1∶2000. Secondary antibody binding was detected using DAB substrate.

### Incubation of macrophages with different treatments

PMA-differentiated RAW264.7 cells were put into 24-well flat bottom tissue culture plates at a density of 1×10^5^ cells per well. The cells were incubated at 37°C. Cells were washed, and medium was replaced 4 hours before treatment. Cells were incubated with BCG-PPD (10 µg/ml), or mixtures of PPD (10 µg/ml) and MPT64 at different concentrations from 10 to 20 µg/ml, respectively. To control the nonspecific effects of the GST tag itself, control cells were incubated with GST protein (20 µg/ml). Furthermore, the groups included treatment with heat-inactivated MPT64 (20 µg/ml) because the MPT64 was produced in *E. coli.* The different groups were incubated at 37°C and 5% CO_2_ for up to 16 hours before the apoptosis assay.

### Apoptosis assays

The apoptosis assay was performed with an Annexin V-FITC apoptosis detection kit (BD Bioscience). The treated macrophages were collected after cold PBS washing and digestion with trypsin at a concentration of 0.25%. After washing twice, the cells were resuspended in binding buffer. An aliquot of 500 µl was removed and mixed with 5 µl of Annexin V-FITC and 5 µl of Propidium iodide (PI). The mixture was incubated for 10 min at room temperature in the dark. Finally, the cells were analyzed by flow cytometry. For each condition, 10,000 events were collected, and the percentage of AnnexinV-positive and PI-negative cells was determined. The experiments were repeated three times, and the average values and standard deviations were calculated.

### Western blots for detecting the expression of bcl-2 in macrophages

Cell lysates were centrifuged for 10 min at 4°C, and the supernatant was obtained. The protein concentration was determined using the Lowry method, and 30 µg protein was used for 15% SDS-PAGE. The separated proteins were electrophoretically transferred onto polyvinyldene difluoride (PVDF) membranes. After blocking with 5% non-fat milk in PBS, the membranes were probed overnight at 4°C with mAb against Bcl-2 at a dilution of 1∶1000. The bound antibodies were detected by incubation for 1 h at 37°C with horseradish peroxide-conjugated secondary antibodies (dilution 1/1000) for 1 h. Reactive bands were detected using an ECL chemiluminescence system (Santa Cruz).

### Quantitative Real-Time Polymerase Chain Reactions

Quantitative real-time polymerase chain reactions (QRT-PCR) were performed to confirm the differential expression of selected genes among the different groups. Primer sequences were designed based on alignments of candidate gene sequences. RNA samples were treated with DNA-free (Takara), following the manufacturer's instructions, to remove contaminating genomic DNA. Total RNA was reverse-transcribed using SuperScript II (Takara). Five microliters of the reverse transcription reaction was added to 45 µl of SYBR green PCR master mix (Takara). Forty cycles of amplification, data acquisition and data analysis were performed.

The sequences of the specific primers were as follows: 5′gtg aga agt gag gga cct tta tg 3′(forward) and 5′cac tca tta gcc ata tcc aac ttg 3′ (reverse) for bcl-2; 5′atg gag ctg cag agg atg 3′ (forward) and 5′tgt cca gcc cat gat ggt tc3′ (reverse) for bax; 5′cgg ttc cga tgc cct gag gct ctt 3′ (forward) and5′ cgt cac act tca tga tgg aat tga 3′ (reverse) for β-actin; 5′ tag ctt atc aga ctg atg ttg a 3′ for miRNA21.The PCR reaction program for bcl-2 was as follows: 1 min at 95°C, followed by 40 cycles of 15 sec at 95°C, 15 sec at 57°C and 15 sec at 72°C. The PCR reaction program for bax amplification was as follows: 1 min at 95°C, followed by 40 cycles of 15 sec at 95°C, 15 sec at 57°C and 15 sec at 72°C. The PCR reaction program for miR21 amplification was as follows: 1 min at 95°C, followed by 40 cycles of 15 sec at 95°C, 15 sec at 57°C and 15 sec at 72°C.

### Luciferase Reporter Assays

The primers for bcl-2 3′UTR were designed as follows: (1) bcl2 3′UTR wild type primers (bcl2-utr-wt), forward primer, 5′cca ctg aga ctt ccc tgc tga 3′, and reverse primer, 5′ tgg gca cta cct gcg ttc 3′; (2) bcl2 3′UTR mutant primers (bcl2-utr-mut), forward primer, 5′ttc acg tac caa ttg tgc cga g 3′, and reverse primer, 5′ tgg gca cta cct gcg ttc 3′. The amplified sequences were inserted into the SpeI and HindIII sites of the pMIR-report™ luciferase vector, respectively.

RAW264.7 macrophages were seeded at 4×10^4^ cells/well in flat-bottom tissue culture plates and co-transfected with 800 ng of pMIR-report-bcl2-utr-wt, 80 ng of pRL—TK (Promega) and 20 pmol miRNA21 by lipofectamine™ 2000 (Promega). The recombinant mutant plasmid pMIR-report-bcl2-utr-mut was also transfected using this method. miRNA-335 was used as control miRNA. The luciferase assay was performed using a Luciferase detection kit (Promega), as previously described [12]. Three wells were used for each Luciferase Assay sample.

### Chromatin immunoprecipitation (ChIP) assays

ChIP experiments were performed in RAW264.7 cells using the ChIP assay kit (Millipore), as previously described [9], and NF-κB rabbit Ab (C-20, Santa Cruz Biotech, Inc.). Primers specific to the mouse miR-21 promoter are listed in Table 1.

**Table 1:** Primers for NF-kB in ChIP assay.

sites	sense primers	anti-sense primers
site1	5′ AGTTTCTGGGCAAACATCCA 3′	5′ CACCTCCCCCCAGCCAG 3
site2	5′ GTGCCTCCCCAATGTGCTAA 3′	5′ AAAGAAACTGCCCTCCCTCTC 3′
site3	5′ GATAAGGATGACGCAGGG 3′	5′ AAAGAAACTGCCCTCCCTC 3′
site4	5′ AGTGGTGATAAATGTGGGACTT 3′	5′ CATTTGGGAGAAAGTAGGAGA 3′
site5	5′ GGAGGGCTCTAACCAGGAA 3′	5′ GGGTGTAGACACCTTACCTTAAC 3′

### siRNA blockade experiments

siRNA specific for bcl-2 or NF-κB was synthesized by GenePharma Co. The siRNA sequences for bcl-2 [13] were as follows: sense strand, 5′-AAGUACAUACAUUAUAAGCUG-3′; antisense strand, 5′CAGCUUAUAAUGUAUGUACUU-3′.The siRNA sequences for NF-κB p65 [14] were as follows: sense strand, 5′-AAGAAGCACAGAUACCACCAA-3′; antisense strand,5′ –UUGGUGGUAUCUGUGCUUCUU-3′.The siRNAs were dissolved in RNase-free water to a final concentration of 20 µM.

One day before transfection, RAW264.7 cells were seeded in 2 ml of complete medium without antibiotics in a 6-well plate, so the macrophages could reach 30–50% confluency for transfection. The transfection was performed according to the manufacturer's instruction. A total 5 µl of siRNA was added to 250 µl of Opti-MEM (Invitrogen), and 2.5 µl of Lipofectamine 2000 (invitrogen) was diluted in the same amount of medium. After incubation for 5 min at room temperature, the diluted siRNA was mixed gently with diluted Lipofectamine 2000 and incubated for 20 min at room temperature. The mixture was then added to the plates with 1.5 ml of serum-free, antibiotic-free medium.

After 16 h of transfection, apoptosis of RAW264.7 cells was detected. The cells were harvested, and total RNA was isolated with Trizol reagent (Takara), according to the manufacturer's instructions. Total RNA was reverse-transcribed using SuperScript II (Takara), followed by dilution of the reverse transcription products to 1∶20. Five microliters of the reverse transcription reaction was added to 45 µl of SYBR green PCR master mix (Takara). The sequences of the specific primers were: 5′gtg aga agt gag gga cct tta tg 3′(forward) and 5′cac tca tta gcc ata tcc aac ttg 3′ (reverse) for bcl-2; 5′cgg ttc cga tgc cct gag gct ctt 3′ (forward) and5′ cgt cac act tca tga tgg aat tga 3′ (reverse) for β-actin. The PCR reaction program was as follows: 1 min at 95°C, followed by 40 cycles of 15 sec at 95°C, 15 sec at 57°C and 15 sec at 72°C. The levels of individual mRNA transcripts relative to the control were calculated using 2^−△△ct^. The experiment was repeated three times.

### Statistical analysis

All data are represented as the means±SEM from three separate experiments.The difference between two groups was analyzed using Student's t-test.Differences were considered significant at *P*<0.05.

## Results

### Expression and purification of MPT64 recombinant protein in *E. coli*

**Figure 1:**
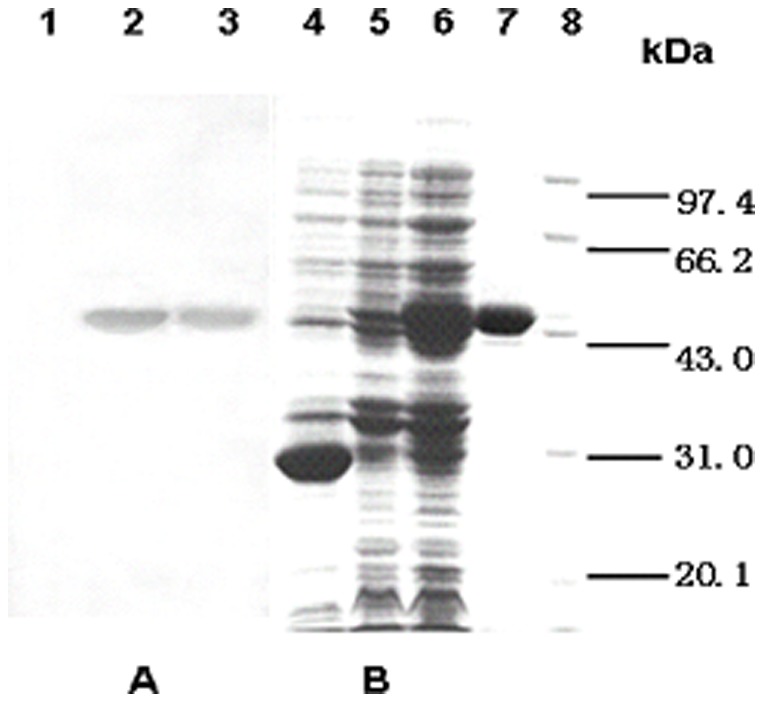
SDS-PAGE electrophoresis and immunoblotting of recombinant MPT64 protein. (A) Proteins were transferred to nitrocellulose and probed with antibody specific for MPT64, and a final detection was made using DAB substrate. (B) Proteins were stained with Coomassie Briliant Blue. Lane 1: crude lysate of *k802* transformed by pGEX5T after induction. Lane 2: the expression product of *k802* transduced with pGEX5T-MPT64. Lane 3: purified MPT64 protein. Lane 4: crude lysate of *k802* transformed by pGEX5T after induction. Lanes 5/6: the expression product of *k802* transduced with pGEX5T-MPT64, before or after induction. Lane 7: purified MPT64. Lane 8: protein markers.

The expression of the gene encoding MPT64 protein from the pGEX5T vector in *E. coli k802* resulted in a GST fusion protein with a molecular mass of approximately 50 kDa after IPTG induction. The GST fusion protein, in contrast to GST, could bind the MPT64-specific antibody (Fig. 1). This result indicated that the purified MPT64 protein was specifically recognized by its antibody. The LPS concentration in the final MPT64 recombinant protein was approximately 10 EU/mg, as measured by a Limulus Assay.

### MPT64 inhibits apoptosis induced by BCG-PPD

**Figure 2:**
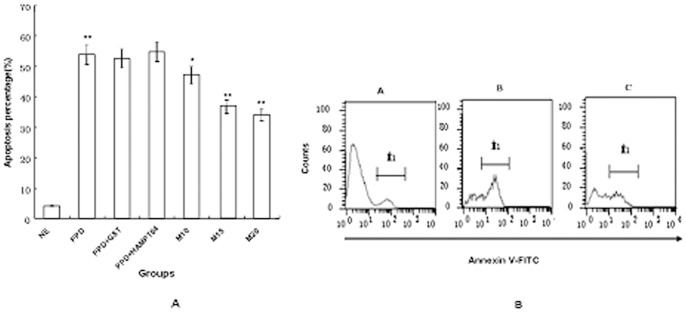
Apoptosis inhibition of macrophages by MPT64 protein. PMA-differentiated RAW264.7 cells were put into 24-well flat bottom tissue culture plates at a density of 1×10^5^ cells per well. Then, RAW264.7 macrophages were incubated with PBS, BCG-PPD (10 µg/ml) or a mixture of PPD-MPT64 at a different concentrations for 16 h. (A) Apoptosis was detected by measuring the membrane exposure of PS using annexin V by flow cytometry, and the results were analyzed. **Fig. 2A** Significant differences of the apoptosis percentages. *P<0.05, the PPD group vs the MPT64 (10 µg/ml) group; ** *P*<0.01, the PBS group vs the PPD group, and the PPD group vs the MPT64 (15 µg/ml or 20 µg/ml) group. The apoptosis percentage is not significantly different for the GST group or heat-treated MPT64 protein compared with the PPD group. **Fig. 2A** Ne, the PBS group; HAMPT64, heat treated MPT64; M10, MPT64 at 10 µg/ml of concentration; M15, MPT64 at 15 µg/ml of concentration; M20, MPT64 at 20 µg/ml of concentration. **Fig. 2B** A: The PBS group. B: The PPD group. C: The MPT64 (15 µg/ml) group. Data shown are representative of three independent experiments.

As reported, BCG-PPD could induce apoptosis of RAW264.7 after 16 h of incubation, with a higher apoptosis percentage than that observed for the PBS control group (*P*<0.01). Interestingly, the apoptosis percentage in the PPD and MPT64 combination group was significantly lower than that of the PPD treatment group. This inhibition was dose-dependent, with a maximum inhibition at approximately 15 µg/ml concentration (Figs. 2A and 2B). The apoptosis percentage induced by GST protein was similar to that observed in PPD-treated cells. This result indicated that the GST tag was unlikely to contribute to apoptosis inhibition.

To examine whether other possible contamination of the purified protein might contribute to apoptosis inhibition by MPT64, MPT64 fusion protein was heat-treated before its addition to cells. As shown in Fig. 2A, the heat-treated MPT64 protein did not contribute to apoptosis inhibition. These findings indicate that the MPT64 protein could indeed inhibit the apoptosis induced by BCG-PPD.

### Apoptosis inhibition by MPT64 is related to bcl-2 up-regulation

**Figure 3:**
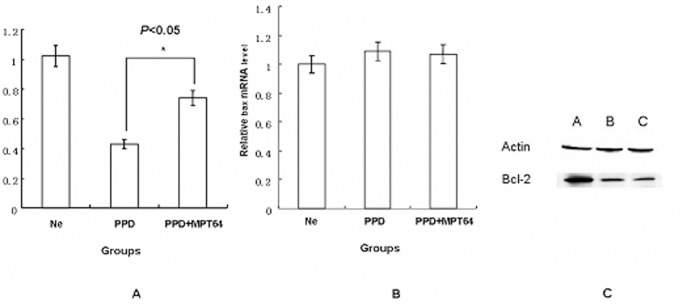
Apoptosis-related gene expression after MPT64 treatment. PMA-differentiated RAW264.7 macrophages were incubated with PBS, BCG-PPD (10 µg/ml), or a PPD-MPT64 (15 µg/ml) mixture for 16 h. Then, the bcl-2 mRNA (3A) and bax mRNA levels were detected by real-time PCR (3B) or Western-blot (3C) for the expression of bcl-2. For the Western-blot, reactive bands were detected by an ECL chemiluminescence system.*P<0.05, the PPD group vs the PPD-MPT64 (15 µg/ml) group. **Fig. 3C** A: The PBS group. B: The MPT64 (15 µg/ml) group. C: The PPD group. Data shown are representative of three independent experiments.

As previously reported, apoptosis of macrophages is related to the bcl-2 gene family during *Mycobacterium tuberculosis* infection [15]. To determine whether this family was involved in apoptosis inhibition by MPT64, primers for bcl-2 and bax were designed, and the mRNA levels were analyzed by real-time RT-PCR. As shown in Figs. 3A and 3B, the bax mRNA level is similar in the PBS group, the PPD group and the PPD-MPT64 mixture group. In contrast, the bcl-2 mRNA levels changed significantly among the three groups. The bcl-2 mRNA level was significantly lower in the PPD group compared with the PBS group (*P*<0.01). Furthermore, the bcl-2 mRNA level was significantly increased in the PPD-MPT64 mixture group (*P*<0.05) compared with the PPD group.

We next determined the bcl-2 protein level in the different groups using Western-blots. As shown in Fig. 3C, the protein levels are consistent with the results of quantitative real-time PCR; the bcl-2 expression level was significantly higher in the PPD-MPT64 group than that in the PPD group.

Taken together, these results suggest that the apoptosis inhibition is due to the increased bcl-2 expression.

### Inhibition of the anti-apoptosis activity of MPT64 by bcl-2 siRNA

**Figure 4:**
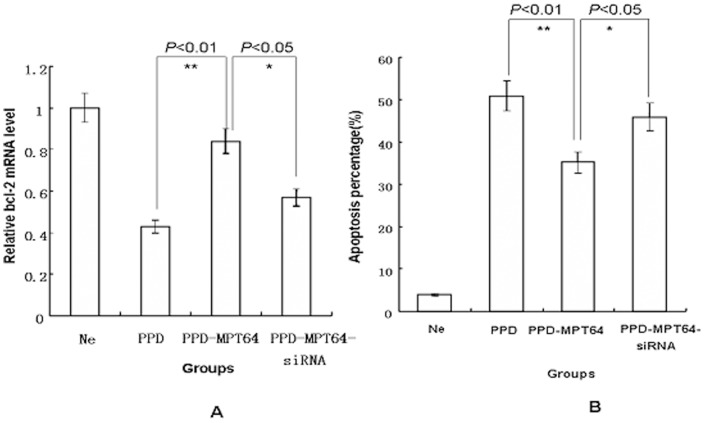
MPT64 anti-apoptosis activity reduced by specific siRNA blockade for bcl-2. siRNA for bcl-2 was synthesized and transfected into differentiated RAW264.7 cells. The cells were divided into four groups: a PBS group, a PPD group, a PPD-MPT64 (15 µg/ml) group, and a PPD-MPT64 (15 µg/ml)-siRNA group, After 16 h of transfection, the mRNA level of bcl-2 (4A) was detected by real-time PCR, and the apoptosis percentage (4B) was analyzed by flow cytometry. Fig. 4A, *P<0.05, the PPD-MPT64 (15 µg/ml)-siRNA group vs the PPD-MPT64 (15 µg/ml) group Fig. 4B, *P<0.05, the PPD-MPT64 (15 µg/ml)-siRNA group vs the PPD-MPT64 (15 µg/ml) group. Data shown are representative of three independent experiments.

To further confirm the effect of bcl-2 during the anti-apoptosis activity of MPT64, siRNA for bcl-2 was synthesized and transfected into RAW264.7 cells. The cells were divided into four groups: a PBS group, a PPD group, a PPD-MPT64 (15 µg/ml) group and a PPD-MPT64 (15 µg/ml)-siRNA group. After 16 h of transfection, the mRNA levels of bcl-2 and the apoptosis percentages were measured. As shown in Fig. 4A, the bcl-2 mRNA in the PPD-MPT64 (15 µg/ml)-siRNA group was similar to that of the PPD group. However, as shown in Fig. 4A, the bcl-2 mRNA in the PPD-MPT64 (15 µg/ml)-siRNA group was significantly lower than that of the PPD-MPT64 (15 µg/ml) group (*P*<0.05). Moreover, the apoptosis percentage was significantly increased in the PPD-MPT64 (15 µg/ml)-siRNA group compared with the PPD-MPT64 (15 µg/ml) group (*P*<0.05). Hence, MPT64 lacked anti-apoptotic activity in the absence of bcl-2.

### miR-21 up-regulates the expression of bcl-2

**Figure 5:**
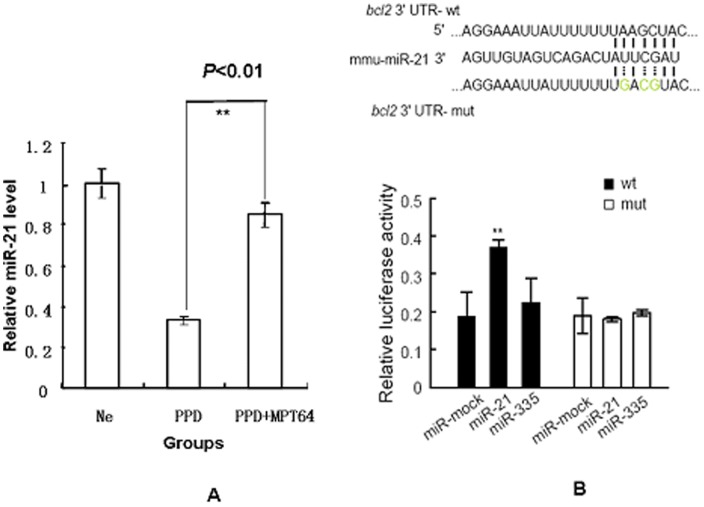
The control effect of miRNA21 on bcl-2 after MPT64 treatment RAW264.7. Macrophages were treated as shown in Fig. 2. The miRNA21 level was analyzed by real time-PCR (5A). **P<0.01, the PPD group vs the PPD-MPT64 (15 µg/ml) group. The control effect of miRNA21 on bcl-2 was also analyzed by relative luciferase activity in RAW264.7 cells (5B). The wild-type or mutated type of bcl-2-utr was cloned into a pMIR-reportTM luciferase vector. Then, the recombinant plasmids and miR21 were transfected into RAW264.7 cells. The relative luciferase activity was detected. miR-mock and miR335 were used as control miRNAs. *P<0.01, the miR21 group vs the miR-mock group after the transfection by wild-type bcl-2-utr. Data shown are representative of three independent experiments.

We next examined the mechanisms by which bcl-2 inhibited apoptosis. It has been reported that miR-21 also promoted dendritic cell (DC) apoptosis by targeting Bcl-2 after BCG vaccination [16]. We thus measured the miRNA21 level by RT-PCR. Interestingly, the results showed that the miRNA21 level was significantly higher in the PPD-MPT64 mixture group than it was in the PPD group (*P*<0.01) (Fig. 5A). This result indicated that the miRNA-21 expression level increased in macrophages after MPT64 treatment.

To assess whether miR-21 can directly alter the expression of bcl-2 in vitro, a fragment of the 3′-UTR of bcl-2 mRNA containing the wild type or mutated putative miR-21 binding sequence was cloned into a luciferase reporter construct, as either pMIR-report-bcl2-utr-wt (wild type, WT) or pMIR-report-bcl2-utr-mut (mutated type, MUT), respectively. The luciferase reporter assay in RAW264.7 cells demonstrated that miR-21 could effectively improve the luciferase activity of pGL3-bcl2-wt (WT) (Fig. 5B, WT). The construct pMIR-report-bcl2-utr-mut (MUT) was used to repeat the luciferase assay experiments in macrophages cells, and it showed that mutating the seed region for miR-21 in the pMIR-report-bcl2-utr-wt plasmid abrogated its regulatory activity (Fig. 5B, MUT). Co-transfection of the control miR-335 did not change the luciferase activity. These results indicate that miR-21 may up-regulate bcl2 expression through a binding site at the 3′-UTR of bcl-2.

### miR-21 is directly regulated by the transcription factor NF-κB

**Figure 6:**
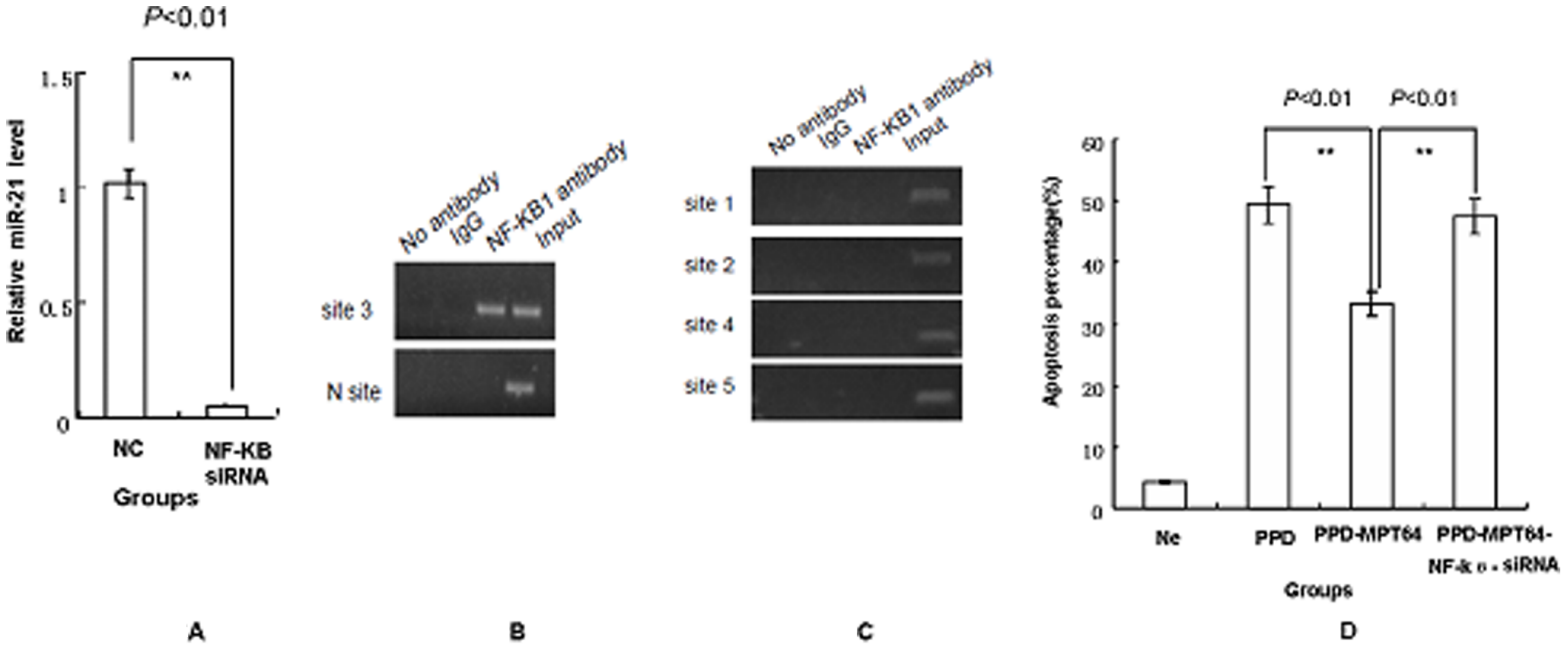
miR21 is directly regulated by NF-kB. Real-time PCR results for miR-21 levels were performed with siRNA against NF-κB. After the transfection of siRNA against NF-κB, the miR-21 level was detected by real-time PCR (6A). **P<0.01, the siRNA treatment group vs the non- siRNA treatment group. Then, the apoptosis level was analyzed by flow cytometry (6D). **P<0.01, the siRNA treatment group vs the non- siRNA treatment group. Five primers were designed according to a software analysis, and ChIP assays were performed in RAW264.7 cells to explore possible binding sites. A specific band was observed for primer for site 3 (6B). No specific band was observed for the primers for sites 1, 2, 4 and 5 (6C). Negative controls were incubated without primary antibody. The experiment was repeated three times.

We next determined how the transcription of miR-21 was controlled. First, we predicted the candidate transcription factor for miRNA21 by bioinformatics software. The analysis showed that NF-κB was one of the most likely transcription factors for miRNA21 because it has five possible binding sites at miRNA21. Furthermore, it has been reported that *Mycobacterium tuberculosis* can activate NF-κB to prevent apoptosis of host cells. Then, a ChIP experiment was performed to explore whether NF-κB was a transcription factor for miRNA-21. We designed five PCR amplicons to evaluate the presence of putative binding sites in the chromatin immunoprecipitates. The experiments in RAW264.7 revealed that NF-κB specifically binds to the putative promoter of miR-21 at site3 (Fig. 6B). Furthermore, we tested whether miR-21 expression could be reduced by siRNA against NF-κB (Fig. 6A). An siRNA sequence [17] was synthesized and transfected into RAW264.7 cells. Forty-eight hours later, the level of miR-21 was detected by real-time PCR. The results showed that siRNA against NF-κB could inhibit the miR-21 levels. Taken together, the results suggest that the up-regulation of miR-21 is due to the increased NF-κB expression in RAW264.7 after MPT64 treatment.

To confirm the role of NF-κB in MPT64 treatment, the apoptosis of RAW264.7 cells by an NF-κB-specific siRNA treatment was assayed. The PMA-differentiated RAW264.7 cells were divided into four groups: a PBS group, a PPD group, a PPD-MPT64 group and a PPD-MPT64-NF-κB siRNA group. NF-κB siRNA was transfected into RAW264.7 cells using the method described above.

After 16hours of treatment, the apoptosis percentage was detected by flow cytometry. As shown in Fig. 6D, the apoptosis percentage in the NF-κB siRNA treated RAW264.7 cells of the MPT64 treatment group increased significantly compared with the PPD-MPT64 group (*P*<0.01). Hence, this experiment demonstrated that NF-κB siRNA could abrogate the anti-apoptosis activity of MPT64.

## Discussion

After infection, *Mycobacterium tuberculosis* lives and replicates in host macrophages. To evade the host immune response, *Mycobacterium tuberculosis* uses strategies to resist apoptosis of the macrophages. Although many studies have examined the occurrence of anti-apoptosis in macrophages that have ingested virulent mycobacteria, studies to identify the molecules involved are rare. So far, only a few *Mycobacterium tuberculosis* components that resist apoptosis of macrophages have been found. These include SecA2, Rv3654v, Rv3655c and protein kinaseE (PknE). A better understanding of host-pathogen interactions will enable the development of novel therapeutics and shorten treatment times.

In this study, we demonstrate that the secreted protein MPT64 of *MTB*, also known as Rv1980c, is anti-apoptogenic for RAW264.7. MPT64 is deleted from nearly all BCG strains [18] and is only expressed and secreted from actively growing cells [19]. Loss of this region has been correlated with a drop in virulence. MPT64 may thus function as a mycobacteria-specific virulence factor. In our study, we explored the possible effect of MPT64 on macrophages.

It has been reported that PPD from different strains of mycobacterium has different effects on macrophages. PPD from BCG can cause apoptosis, and PPD from virulent strains of Mycobacterium mainly cause necrosis [20]. Our study showed that BCG-PPD could induce apoptosis of RAW264.7. However, when RAW264.7 macrophages were treated with MPT64, the percentage of apoptosis decreased significantly. This result indicated that MPT64 could inhibit the apoptosis of RAW264.7 macrophages induced by BCG PPD. Our result is identical to that of a previous study, which showed that in tuberculosis granulomas, the expression level of MPT64 is inversely related to the amount of apoptosis of host immune cells [21]. Hence, MPT64 is indeed a virulence factor during infection by *MTB*.

We next sought the potential mechanism behind MPT64 apoptosis inhibition. It has been reported that during the infection of *Mycobacterium tuberculosis*, TNF-a and nitric oxide are the most important pro-apoptotic mediators [22]. However, the bcl-2 family plays an important role in inhibiting apoptosis of macrophages [15], [23]. In our study, we show that MPT64's anti-apoptotic property was partially dependent upon bcl-2 activity, with an increase in the mRNA and protein levels in the MPT64 treatment group compared with the PPD group. Our result is not identical to that obtained in Tehmina's study. He found that there was a negative correlation between MPT64 expression and apoptotic markers, such as Fas and FasL. The apoptotic molecules in our study are different from Tehmina's, which may correlate with the different cell models used.

The microRNAs are a class of endogenous, noncoding RNAs that act post-transcriptionally to target mRNAs [24]. They can regulate target RNA either by repression or by promotion [25]. Recent studies have revealed the central role of miRNAs in innate immune responses to pathogens and a variety of stimuli [26], [27]. During infection, some microorganisms can regulate apoptosis of host immune cells by miRNAs. During infection by Epstein-Barr virus, BART miRNA can regulate the expression levels to inhibit the apoptosis of B cells [28]. MiRNA21 can down-regulate bcl-2 and promote the apoptosis of dendritic cells [16]. miRNA155 is required for *Mycobacterium bovis* BCG-mediated apoptosis of macrophages [29]. In our study, miRNA21 was found to positively control bcl-2 gene expression and inhibit the apoptosis of macrophages after MPT64 treatment. Recently, miR-21 was also shown to be involved in inflammatory responses [30]. In most reports, miRNA21 represses the target gene level. However, our study showed that a higher level of miR21 can up-regulate the bcl-2 level and inhibit the apoptosis of macrophages. This result is similar to Dong's report, which showed that miR21 can up-regulate bcl-2 levels and resistance to apoptosis in pancreatic cancer [31]. Those different regulation effects of miRNA21 may correlate with different cell models.

NF-κB activation has been shown to enhance immunity against some microbial pathogens [32] through induction of an inflammatory response. However, specific NF-κB-mediated pathways may promote the survival of bacterial pathogens. *Escherichia coli*, *Mycobacterium tuberculosis* and *Chlamydophila pneumoniae* can activate NF-κB to prevent apoptosis of host cells [33], [34]. *Shigella flexneri* and *Helicobacter pylori* can induce host NF-κB to enhance tissue invasion [35]. In our experiment, we analyzed possible transcription factors and found five possible binding sites for NF-κB. One of these five binding sites was confirmed after CHIP experiments, and this control effect can be inhibited by siRNA against NF-κB. Our result is similar to Loeuillet's research, which showed that NF-κB prevented *MTB*-infected THP-1 cells from apoptosis [36]. In our study, NF-κB activation is related to the bcl2-mediated apoptosis inhibition of macrophages.

In summary, MPT64 can resist the apoptosis of RAW264.7 macrophages by PPD in vitro through an NF-κB-miR21-bcl-2 pathway. Blocking apoptosis provides an environment for the growth and replication of *Mycobacterium tuberculosis*, which can result in the evasion of host immune responses. Thus, MPT64 may be a virulence factor during infection of by *Mycobacterium tuberculosis*. Few functions of MPT64 have been clear, other than its special beta-grasp structure [37].The exact mechanism of MPT64 activity needs further study *in vivo*.
